# Epidemiologic Characteristics of Suicide in Panama, 2007–2016

**DOI:** 10.3390/medicina56090442

**Published:** 2020-08-31

**Authors:** Virginia Núñez-Samudio, Aris Jiménez-Domínguez, Humberto López Castillo, Iván Landires

**Affiliations:** 1 Instituto de Ciencias Médicas, Las Tablas, Los Santos 0701, Panama; vnunez@institutodecienciasmedicas.org (V.N.-S.); arisjimenezdominguez@gmail.com (A.J.-D.); 2 Department of Health Sciences, College of Health Professions and Sciences, University of Central Florida, Orlando, FL 32816, USA; humberto.lopezcastillo@ucf.edu; 3 Department of Population Health Sciences, College of Medicine, University of Central Florida, Orlando, FL 32827, USA; 4 Centro Regional Universitario de Azuero, CRUA, Universidad de Panamá, Chitré, Herrera 0601, Panama; 5 Hospital Joaquín Pablo Franco Sayas, Región de Salud de Los Santos, Ministry of Health, Las Tablas, Los Santos 0701, Panama

**Keywords:** suicide, mortality, rate, Panama, demographics

## Abstract

*Background and objectives:* We aim to describe the demographic characteristics associated with suicide in Panama, to estimate the suicide mortality rate and years of potential life lost (YPLL) to suicide, and to explore the correlation of suicide rates with the Multidimensional Poverty Index (MPI). We present a descriptive retrospective epidemiological report of suicide-related mortality (Panama, 2007–2016). *Materials and Methods:* Data were matched-merged to calculate unadjusted suicide mortality rates (overall, and by sex, age groups, and administrative region), YPLL, and coefficients (*r*) for the correlation of MPI and suicide rates. *Results:* There were 1475 deaths by suicide (86% among men, 47% between 20 and 39 years). The average mortality rate was estimated at 3.91 per 100,000 population with an average YPLL rate of 3.79 per 1000 population. There was a statistically significant trend to reduce YPLL over time (*r* = −0.93; *p* *<* 0.001). Exploratory analyses did not show a significant correlation between the MPI and suicide rates. Our study showed a 6:1 male-to-female ratio of suicide, mostly affecting the age groups of 20–29 and over 80 years. Conclusions: Exploratory analyses on the correlation of the MPI and the suicide rates did not achieve statistical significance, and alternative explanations, such as access to pesticides and alcohol, were further explored to inform potential interventions.

## 1. Introduction

Suicide—Death caused by self-directed injurious behavior with any intent to die as a result of the behavior—Is a worldwide public health issue [1]. Suicidal behavior is a continuum that includes ideation, planning, attempt, and completion [2,3]. A recent study [4] used 2016 worldwide data to estimate that 817,000 persons committed suicide, representing 1.49% of total deaths with an overall mortality rate of 11.1 per 100,000 population. That year, there were 34.6 million years of potential life lost (YPLL) to suicide—2.18% of all YPLL for an age-standardized rate of 458.4 YPLL to suicide per 100,000 population. Although the age-standardized mortality rate for suicide decreased by 32.7%, in 2016, there was a 6.7% increase in the total number of deaths over the prior 27 years [4]. It is estimated that 75.5% of all deaths by suicide occur in low- and middle-income countries [5], and suicide is the second cause of death among 15- to 29-year-olds.

The Pan-American Health Organization [6] reports that, every year, there are some 65,000 deaths by suicide in Latin America, with an overall age-adjusted mortality rate of 7.26 per 100,000 population. The age-adjusted mortality rate in the Latin American countries in 2005–2009 ranged from 1.01 (Peru) to 14.4 (Uruguay) per 100,000 population [6].

The Republic of Panama is a transcontinental country in Central and South America, with Panama City serving as the capital, government-see, and largest city—Home to nearly half the country’s 4 million population [7]. Panama is among the only three high-income–economy countries in Latin America, along with Chile and Uruguay [8]. In 2018, Panama ranked 66th in the world in terms of the Human Development Index [9] and was the seventh most competitive economy in Latin America by the World Economic Forum’s Global Competitiveness Index [10]. Panama is organized into 10 provinces—The main regional geographic circumscription—And 5 comarcas created by special law regimes for geographic, ethnic, and cultural criteria [11,12].

In Panama, mortality due to external causes, including suicide, has steadily increased in the last decade, and, in 2018, it represented the third overall cause of death in the country [13,14]. A recent study [15] found that the annual percent change (APC) of suicide in Panama between 2001 and 2016 declined for women (mean APC of −4.8), while the decline for men was only observed starting in 2006 (mean APC −6.9).

Several genetic, psychological, and psychiatric risk factors have been described in association with suicide [16,17]. For example, people with a history of substance use disorder (SUD) have been found at increased risk of suicide behavior, to the point that guidelines for treating people with SUD include asking for a history of suicide ideas and attempts [2]. Worldwide and in Latin America, comorbidity of SUD and other psychiatric disorders, such as schizophrenia, increases the risk of suicidal behavior [18]. Among the SUD, alcohol use, misuse, and abuse is of particular concern. It has been noted that, compared to women, men start alcohol use at an earlier age, develop more regular consumption, and develop higher dependence [19]. Negative life events, such as sexual abuse during childhood [20] and housing instability [21], have been found to be triggers of suicide attempts, especially among the young and those without mental disorders [22].

Sociocultural factors are constantly described both as an independent risk factor or as an element within the multifactorial causality of suicide. In Latin America, where most of the countries are classified as low- or middle-income economies, there have been reports associating suicide and poverty. A time-series analysis in Colombia found a significant negative correlation (*r* = −0.91; *p* < 0.001) between the gross domestic product per capita and the suicide rates [23]. However, a study based on Colombia’s 2015 National Mental Health Survey found no correlation between poverty levels, measured through the Multidimensional Poverty Index (MPI), and suicidal ideation [24]. The MPI identifies multiple deprivations at the household and individual level in health, education, and standard of living. Even though it shows a great deal of heterogeneity within Latin America, the MPI is considered a good quantitative measure of the poverty level within each country and its regions [25].

Currently, there is a dearth of epidemiological studies on suicide in Panama. Thus, we aim to analyze ten years of data (2007–2016) to describe the demographic characteristics associated with suicide in Panama, to estimate the unadjusted suicide mortality rate and YPLL to suicide, and to explore the correlation of suicide rates with the MPI as a multidimensional measure of structural poverty.

## 2. Methods

This is a descriptive retrospective epidemiological study of suicide-related mortality from 2007 to 2016 in the Republic of Panama.

### 2.1. Data Sources

We obtained the raw data from the suicide registries of the Instituto Nacional de Estadística y Censo (INEC) [13]. This database contained the following variables for each case of suicide reported during the study period: year, sex, age, and administrative region where suicides occurred (i.e., 10 provinces and 5 comarcas of the Republic of Panama as of 2016). Age was grouped into eight brackets: 10–19, 20–29, 30–39, 40–49, 50–59, 60–69, 70–79, and 80 and more years. We also obtained Panama’s population estimates published by the INEC for the respective study years [13].

Data on life expectancy at birth, overall and by gender, were downloaded from The World Bank’s DataBank (https://data.worldbank.org/indicator/SP.DYN.LE00.IN?locations=PA) and data for the MPI were obtained from the 2017 MPI Panama Report [25].

### 2.2. Analyses

Data were analyzed in SPSS Statistics for Windows v. 23 (IBM Corp.; Armonk, NY, USA).

Demographic characteristics were reported using descriptive statistics (i.e., frequencies and means) for the study period. Unadjusted suicide mortality rates (per 100,000 population) were calculated for each year, overall and by gender and age group, dividing the number of cases by corresponding total.

To estimate the YPLL, we used the INEC data for suicide cases and population estimates overall and by gender during the study period. For each group (i.e., males, females, and total), we calculated YPLL as the difference between the Panamanian life expectancy for their sex and age at death that year and the mean age of the population group [26]. This estimate should be interpreted as “the average time a person would have lived had he or she not died prematurely [and] is used to help quantify social and economic loss owing to premature death, and it has been promoted to emphasize specific causes of death” [27].

Correlation analyses between mortality rates and the MPI for the country and the administrative regions were conducted using Pearson’s product–moment correlation coefficient (*r*), and the explanation of variability in the model used the coefficient of determination (*R*^2^).

## 3. Results

### 3.1. Demographic Characteristics

**Figure 1:**
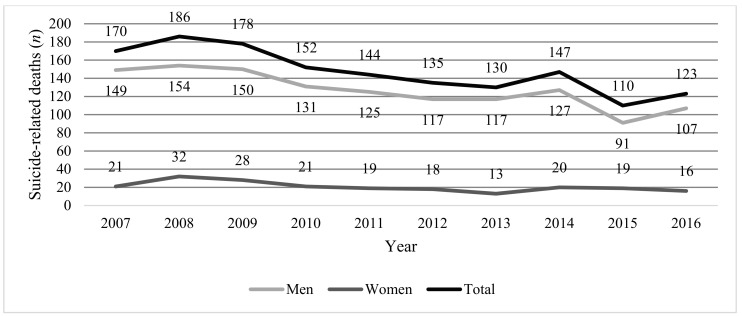
Time series of suicide-related deaths in Panama, total and by sex, 2007–2016. Note: Total may be higher than the sum of males and females because it accounts for suicide-related deaths where sex was not specified.

The trend over time of total deaths by suicide in Panama are presented in Figure 1, and Table 1 shows the number of suicide-related deaths by sex and the male-to-female ratio of these cases. Between 2007 and 2016, there were 1475 reports of deaths by suicide in the Republic of Panama, 86% of which were among men and 47% of which occurred between ages 20 and 39. The overall male-to-female ratio was 6:1, with a peak of 12:1 observed in the 60-to-69-years age group and the lowest ratio of 2:1 observed in the 10-to-19-years age bracket.

### 3.2. Unadjusted Suicide Mortality Rates

Table 2 shows the unadjusted suicide mortality rates (per 100,000 population) for the study period. During the 2007–2016 decade, the overall unadjusted suicide mortality rates showed a significant trend to decline over time (*r* = −0.78; *p* = 0.007) and ranged from 3.1 in 2015 to 4.7 in 2008. For males, crude suicide mortality rates ranged from 4.6 in 2015 to 8.7 in 2008, while for females, these values were between 0.7 in 2013 and 1.8 in 2008.

Table 3 shows the unadjusted suicide mortality rate (per 100,000 population) trends over the study period, by age brackets. Peak adjusted mortality rates were consistently observed in the 20–29 and 30–39-years and in the 80-years-and-older age groups.

### 3.3. Years of Potential Life Lost

Table 4 presents the detail of YPLL by sex. The overall YPLL per 100,000 population rate shows a significant trend to decline over the decade (*r* = −0.93; *p <* 0.001). Most of the burden of YPLL occurs among males and in the group of 20-to-29-year-olds (not shown in Table 4).

### 3.4. Exploratory Correlation with the Multidimensional Poverty Index

Table 5 shows the geographic distribution of the 10-year average unadjusted suicide-related mortality rates (per 100,000 population) by administrative region. In the provinces, the highest and lowest suicide mortality rates were found in Los Santos (10.27) and Colón (1.86), respectively. In the comarcas, the highest and lowest suicide rates were found in Ngäbe Buglé (6.17) and Emberá Wounaan (0.96), respectively. Of notice, no data were reported for the Madugandí (established in 1996) and Wargandí (established in 2000) comarcas; these cases are most likely included in the neighboring provinces of Panamá, Colón, and Darién.

Correlation analyses between suicide mortality rates and the MPI show an overall nonsignificant Pearson product moment correlation coefficient at *r* = −0.41 (*p* = 0.18) for all the administrative regions. Subanalyses by provinces (*r* = −0.48; *p* = 0.19) and comarcas (*r =* 0.56; *p =* 0.62) show similar results. Of note, however, is that while the correlation tends to be inverse in the provinces, the comarcas show a direct correlation. Coefficients of determination (*R*^2^) for these analyses show a moderate explanation of suicide rate variability overall (0.16) and by provinces and comarcas (0.31 and 0.23, respectively).

## 4. Discussion

This study used local population and suicide data to describe the demographic characteristics associated with suicide in Panama and to estimate the rates of YPLL to suicide. Additionally, we explored the correlation of suicide rates with the MPI as a multidimensional measure of structural poverty. During the study decade, Panama had a total of 1475 suicide-related deaths in the study period, averaging 148 suicides per year and a 10-year average suicide rate of 3.91 (range 3.11, 4.68) suicides per 100,000 population. These rates were lower than the average 6.4 reported for Central America [4]. However, it is important to highlight that Panama has an estimated subregistry of 14.4%, just over twice the estimated subregistry for the Americas (7.1%) [6].

Since 2007, Panama’s Ministry of Health has made efforts to prevent and control suicide, establishing the National Intersector Commission for the Prevention and Control of Suicidal Behaviors and Other Forms of Violence in Panama. This Commission aims to contribute to improve the mental health of the general population, focusing efforts in groups where suicide behavior is most prevalent [28]. Reports from 2015–2018 from the Ministry of Health show a favorable impact in decreasing mortality and YPLL [29].

While we observed that the overall trend over time showed a statistically significant decline, we also noticed that the suicide mortality rates peaked in the 20–29 and 80-and-over age groups. Globally, suicide is the second cause of death in the 15-to-29-year-old age group [5]. There are various reports in the Americas region finding elevated mortality rates at older ages [30,31]. However, globally, suicide-related mortality is not registered within the top ten causes of death in people 70 and over [32]. Worldwide, suicide represents the second cause of death among 15-to-29-year-olds. In Panama, external causes—Which include homicide, suicide, and traffic fatalities—Represent the third cause of death in the 20-to-29-year-old population [14].

The data analyzed show that 86% of the cases were found among males, with an overall male-to-female ratio of 6:1. This ratio was fairly consistent throughout the study period, with a minor decline in 2015 (4.75:1). Gender differences in alcohol use, misuse, abuse, and dependence and their association with suicide behaviors may partially explain some of the study findings. A recent publication showed that men have an earlier start of alcohol use, with more regular use, and higher dependence rates. Alcohol-dependent women, on the other hand, had a higher rate of suicide attempts and depression symptoms than males [19]. Worldwide, the suicide-related mortality rate among men is 2.2 times higher than that of women (15.6 vs. 7.0 deaths per 100,000 population, respectively). This disparity is inflated by external causes, which in Panama represents an excess of 52 deaths per 100,000 population for men. These data suggest the need for a suicide-behavior prevention policy, emphasizing gender-specific risk factors.

We did not find a statistically significant association between the MPI and the suicide rates. Possible explanations include the fact that rates were unstandardized and that the MPI might not be capturing socioeconomic variables that are driving suicide rates in Panama. Systematic reviews, however, show an association between suicide and poverty, especially in low- and middle-income countries [33,34,35].

Geographic and socioeconomic patterns within countries may vary the suicide mortality rates [30,36,37]. Our results show heterogeneity in the suicide mortality rates (per 100,000 population) estimated for each administrative region, ranging from 0.96 to 10.26. The central provinces of Los Santos, Herrera, and Veraguas—Which have a larger European descent population—Had the highest suicide rates, more than twice the country’s average. On the other hand, the provinces of Colón, Panamá, Darién, and Bocas del Toro—Which have a larger Afro-Caribbean descent population—Showed the lowest suicide rates. Among the native Panamanian groups, the Ngäbe Buglé people have the highest suicide rates, while the Guna Yala and Emberá Wounaan people have the lowest overall suicide rates. This is consistent with a report using mitochondrial gene pools to determine the descent of modern-day Panamanians [38].

Considering the multicausality of suicide, sociocultural factors should also be considered in light of our findings. The most commonly reported suicide methods in Panama include hanging, use of pesticides, and firearms [39,40]. In Panama, the use of pesticides averages 2.2 kg per capita, which is slightly higher than the 2.0 kg reported for the Central American neighbors [41]. In Panama, the central provinces are characterized by agricultural production, with an increased use and availability of pesticides [42] and the highest reported suicide rates. Moreover, these provinces also have the highest proportion of regular alcohol consumption, with prevalence over 3% in young adults between 18 and 24 years of age [29,43]. Thus, increased availability of pesticides and high rates of alcohol consumption at a young age are sociocultural factors that could explain, at least partially, the increase in suicide rates in these provinces. However, the WHO [5] has established various effective, evidence-based interventions to prevent suicide. Among these strategies, restricting access to common suicide methods have demonstrated to be efficacious in reducing suicide rates [5]. For example, in Sri Lanka, Gunnell et al. [44] demonstrated that import controls for the most toxic pesticides may have an important effect in reducing suicide rates. On the other hand, ideally, treatment of young adults with alcohol use disorders and co-occurring suicidality should follow an integrated protocol that addresses both conditions [45]. Future directions include, thus, strengthening suicide prevention strategies in Panama in alignment with the WHO recommendations by limiting access to pesticides and preventing and treating alcohol use disorders at early ages and by gender-specific risk factors. While these variables make sense within the context of the central provinces in Panama, we cannot rule out the potential role of other variables, such as unemployment rates, socioeconomic status, access to physical and mental health care, and provision of suicide intervention strategies. Moreover, according to the present study, prevention programs intended to prevent suicidal behavior should be implemented among young adults and the elderly.

### Limitations

This study is not void of limitations. First, although INEC makes an outstanding effort to secure the quality of data reports, there is a subregistry of cases [6], no data reports on two comarcas (Madugandí and Wargandí), and the possibility of misclassification of causes of death in the death certificates used as the primary data source. Although there may be several sources for misclassification, stigma related to suicide may play an important role in the subregistry of suicide-related deaths, especially in more conservative, rural areas [46]. The effect of this subregistry would be to underestimate the suicide rates obtained in this study. Second, the suicide rates reported are unstandardized, and the role of population distribution cannot be ruled out. Future efforts could approach the estimation of age- and sex-adjusted suicide mortality rates using standard populations [47,48].

Limitations aside, this is among the first descriptive reports of suicide-related mortality trends in Panama, which shows a distinct pattern when the data are compared with the neighboring Latin American countries and with global data. Data will help in informing interventions to prevent and reduce suicide rates in the country and in the Central American region.

## 5. Conclusions

Our study showed a 6:1 male-to-female ratio of suicide, mostly affecting the age groups of 20–29 and over 80 years. The average suicide mortality rate was estimated at 3.91 deaths per 100,000 population with an average YPLL rate of 3.79 per 1000 population and a statistically significant trend to reduce the YPLL to suicide over time. Exploratory analyses on the correlation of the MPI and the suicide rates did not achieve statistical significance, and alternative explanations, such as access to pesticides and alcohol, should be further explored.

## Figures and Tables

**Table 1:** Suicide-related deaths in Panama by age group and sex, 2007–2016.

Age Group, Years	Suicide-Related Deaths, n (%)			Male-to-Female Ratio
	Total ^a^	Males ^b^	Females ^b^	
10–19	184 (13)	123 (67)	61 (33)	2.01
20–29	396 (27)	342(86)	54 (14)	6.33
30–39	294 (20)	268 (91)	26 (9)	10.31
40–49	206 (14)	179 (87)	27 (13)	6.23
50–59	152 (10)	134 (88)	18 (12)	7.44
60–69	105 (8)	97 (92)	8 (8)	12.12
70–79	75 (5)	67 (89)	8 (11)	8.35
≥80	44 (3)	39 (89)	5 (11)	7.80
**Total**	**1475 (100)**	**1268 (86)**	**207 (14)**	**6.12**

^a^ Percentages calculated from the column total. ^b^ Percentages calculated from the row total.

**Table 2:** Unadjusted suicide mortality rates by sex in Panama, 2007–2016.

	Unadjusted Suicide Mortality Rates (per 100,000 Population)		
Year	Total	Males	Females
2007	4.21	8.52	1.22
2008	4.68	8.65	1.82
2009	4.55	8.28	1.56
2010	3.95	7.11	1.15
2011	3.80	6.68	1.03
2012	3.63	6.15	0.96
2013	3.55	6.05	0.68
2014	4.08	6.46	1.03
2015	3.11	4.56	0.96
2016	3.54	5.28	0.80

**Table 3:** Unadjusted suicide mortality rates (per 100 population) in Panama by age groups, 2007–2016.

	Unadjusted Suicide Mortality Rates (per 100,000 Population) for Age Groups (Years)							
Year	10–19	20–29	30–39	40–49	50–59	60–69	70–79	≥80
2007	2.34	7.48	6.61	8.21	6.92	6.94	6.19	10.51
2008	4.63	9.93	5.76	5.38	5.95	8.37	7.94	6.01
2009	3.81	7.84	8.06	3.86	6.05	9.70	6.69	1.91
2010	2.41	6.45	5.61	6.85	3.55	5.72	5.52	10.93
2011	2.38	6.06	4.47	6.02	4.33	5.51	6.20	10.42
2012	2.35	6.82	4.42	2.94	3.86	4.83	8.53	8.28
2013	2.18	4.18	5.07	4.09	5.15	4.65	7.39	4.74
2014	3.73	6.69	4.15	1.80	3.59	1.34	7.11	6.03
2015	1.28	4.89	4.62	2.54	4.00	2.15	3.04	8.63
2016	2.25	4.52	3.90	3.26	3.61	4.13	6.59	6.87

**Table 4:** Years of potential life lost to suicide in Panama, 2007–2016.

Year	Years of Life Lost			
	Males	Females	Total	Total (per 1000 pop.)
**2007**	10,947.2	1665.17	12,968.5	4.69
**2008**	11,333.2	2544.86	14,221.6	5.04
**2009**	11,059.4	2233.14	13,642.1	4.74
**2010**	9678.7	1679.50	11,677.9	3.97
**2011**	9256.9	1523.55	11,091.0	3.70
**2012**	8686.2	1446.97	10,424.2	3.41
**2013**	8709.2	1047.50	10,063.7	3.22
**2014**	9479.7	1615.14	11,408.7	3.59
**2015**	6811.8	1537.63	8558.7	2.64
**2016**	8032.6	1297.47	9594.1	2.90

Abbreviation: pop., population.

**Table 5:** Correlation analyses for suicide mortality rates (per 100,000 population) and the global MPI by administrative regions in the Republic of Panama, 2007–2016.

Administrative Region	10-Year Unadjusted Suicide Mortality Rate	2018 Global MPI
**Provinces**		
**Bocas del Toro**	3.82	0.179
**Coclé**	5.15	0.079
**Colon**	1.86	0.062
**Chiriquí**	6.06	0.045
**Darién**	3.67	0.146
**Herrera**	6.95	0.017
**Los Santos**	10.27	0.014
**Panamá**	2.79	0.037
**Veraguas**	6.81	0.071
**Comarcas ^a^**		
**Guna Yala**	1.37	0.468
**Emberá Wounaan**	0.96	0.288
**Ngäbe Buglé**	6.17	0.469

^a^ Data were not reported for the Madugandí and Wargandí comarcas. Abbreviation: MPI, Multidimensional Poverty Index.
